# Academic detailing of general practitioners by a respiratory physician for diagnosis and management of refractory breathlessness: a randomised pilot study

**DOI:** 10.1186/s12913-015-0861-9

**Published:** 2015-05-09

**Authors:** Aileen Collier, Debra Rowett, Peter Allcroft, Aine Greene, David C. Currow

**Affiliations:** Discipline, Palliative and Supportive Services, Flinders University, Bedford Park, Adelaide, South Australia; Drug and Therapeutics Information Services, Repatriation General Hospital, Daw Park, Adelaide, South Australia; Southern Adelaide Palliative Services, Repatriation General Hospital, Daw Park, Adelaide, South Australia

**Keywords:** Academic detailing, General practice, Breathlessness, Advanced cancer, Palliative care

## Abstract

**Background:**

Academic detailing (AD; also known as educational visiting) facilitates the translation of evidence into practice and has been widely adopted internationally to facilitate practice change. The potential of AD linked to a specific patient and delivered by a specialist physician to general practitioners has not been evaluated.

This pilot study assessed the feasibility and acceptability of AD on the knowledge and confidence of GPs caring for people with advanced cancer who had breathlessness at the end of life.

**Methods:**

In this randomised controlled pilot, 35 patient/GP dyads were randomised to AD or usual care. Key messages included: ensuring reversible causes were optimally treated; non-pharmacological and pharmacological treatments were considered; and oxygen considered for hypoxaemic patients.

**Results:**

*Acceptability*: The majority of GPs randomised to AD agreed to participate, reporting benefits to practice. The majority of GPs in the control group requested a copy of academic detailing written materials at study completion.

*Feasibility*: AD visits to GPs’ offices could be timetabled reasonably easily, with 24 detailing visits occurring.

*Self-reported knowledge and beliefs*: Ninety two percent of GPs reported the topics covered in the AD sessions were useful, with 83 % reporting an increase in knowledge and confidence. AD sessions resulted in 58 % of GPs reporting a change in their approach to the management of breathlessness. By contrast, 81 % of the usual care group reported low confidence in the management and knowledge of breathlessness.

**Conclusion:**

AD was acceptable and feasible to participating GPs. This pilot supports proceeding to a fully powered study.

## Background

Breathlessness that persists despite maximal therapy of treatable causes affects up to 70 % of people with advanced life-limiting illnesses [1-4]. The sensation of breathlessness creates significant distress for patients and their caregivers [5]. Clinical management guidelines that deal with the management of single aetiologies may be of little help to clinicians when faced with patients who have several factors contributing to their breathlessness at the end-of-life [6]. Further, national and international guidelines for the symptomatic management of breathlessness that persists despite optimal treatment of the underlying causes have changed markedly in the last 15 years [7-10].

A recent Australian population study (Caretrack) highlighted the challenges and evidence to practice gaps existing in Australia [9]. Academic detailing (AD) uses an evidence-based approach that establishes an understanding of a practitioner’s beliefs and behaviours in relation to the issue at hand and then proceeds to tailor up to three key messages in response. There is evidence that AD improves evidence-based prescribing of medicines and other changes in practice are well established and underpinned by robust health service intervention studies [11-13].

Building on existing models of AD [14], this randomised pilot study aimed to determine the feasibility and acceptability of using academic detailing to improve understanding of the current evidence about assessing and treating chronic refractory breathlessness. The model of academic detailing included two unique features that may have widespread relevance to future AD research and practice:

- Linking GP educational visits to a specific patient with the index condition/symptom; and
- Using a specialist physician to provide the detailing visits.

The study also sought any evidence of a change in GPs knowledge or behaviour that could inform calculations for a fully powered randomised controlled trial.

## Methods

This pilot study was a randomised, controlled, non-blinded, parallel group study comparing AD by a specialist palliative care/respiratory physician for general practitioners caring for a specific patient with breathlessness at the time of referral to a specialist palliative care service with usual care.

### Setting and participants

The study took place in Adelaide, South Australia. GPs were recruited after referral of one of their patients with breathlessness to the regional palliative care service. Enrolment required consent of both the patient and his/her GP. The study identified patients with cancer referred to the palliative care service with moderate to severe breathlessness at rest or on minimal exertion. Other inclusion criteria included: able to understand English; an Medical Research Council breathlessness score of equal to or greater than three; considered by their GP to have a life expectancy of more than 2 weeks; and opioid naïve.

### Feasibility and acceptability

Feasibility and acceptability were assessed by investigating GP’s acceptability of the AD including the response rate and level of participation in the discussions related to a specific patient for whom they were providing care. GPs self-reported changes in beliefs, behaviours or confidence about the diagnosis and treatment of refractory breathlessness were assessed using a 21-item questionnaire (appendix 1).

### Randomisation

Patient/GP dyad participants were allocated by block randomisation (1:1) to receive one of the two study arms. Randomisation was performed by a computer random allocation program.

### Analysis

Data were entered into the CareSearch data management system. Recruitment of patient/ GP dyads, the response rate and level of participation in AD by GPs, and evidence of self-reported changes in the beliefs, behaviours or confidence of GPs about the diagnosis and treatment of refractory breathlessness in the intervention group are described.

### Details of academic detailing

Uniquely, the detailer was a palliative medicine and respiratory physician and had a pre-existing relationship with many of the participating GPs, in his usual role of providing specialist consultations. One author (DR), a senior pharmacist and recognised expert in the field of AD, provided training and support to the physician. Two visits were made as part of the protocol at mutually convenient times to provide individualised guidance. AD visits were planned at 2 and 4 weeks following the patient’s enrolment in the study to provide adequate time for the patient to visit their GP and for possible implementation of the new knowledge to occur. Sessions covered the following four evidence-based themes that had been developed from the literature: reversible causes had been considered and optimally managed (level 4 evidence) [15-17]; non-pharmacological measures including walking aids, breathing training and pulmonary rehabilitation had been considered (level 1 evidence) [18]; evidence-based pharmacological interventions include regular, low-dose opioids (level 1 evidence) [19-21]; and the consideration of oxygen if therapeutically indicated (level 1 evidence) [22]. The detailer discussed identification and diagnosis of potentially reversible causes of breathlessness in the specific patient that had triggered participation in the study. Additionally, GPs were provided with supporting written information [6] prepared by the research team, including a copy of the current edition of the Palliative Care Therapeutic Guidelines [13].

Outcome measures

The primary outcome measure was the uptake of key messages by GPs. GPs in both arms of the study completed a 21-item questionnaire compiled by the research team about their knowledge, experience and confidence regarding the management of breathlessness for people at the end of life. GPs in the intervention group completed the questionnaire following the two AD visits. GPs in the control arm completed a modified version of the questionnaire at 10 weeks post enrolment.

Ethics approval was granted from the Repatriation General Hospital’s Human Research Ethics Committee. Patients and their GPs provided written informed consent.

## Results

### Participants

#### GP Characteristics

GPs (n = 35) were recruited from a variety of practice settings including individual and group practices. Clinicians were experienced general practitioners with a median of 28 years since graduation.

#### Patient characteristics

Patients ranged in age between 47 and 88 years. The majority (n = 20) had a diagnosis of lung cancer. Other recorded sites of malignancy included colorectal (3) [23]; other GI tract (1); urological (1) [23]; haematological (1); skin (1); breast (1); prostate (1); pancreas (3); other (3).

#### Participation and response rate

**Fig. 1:**
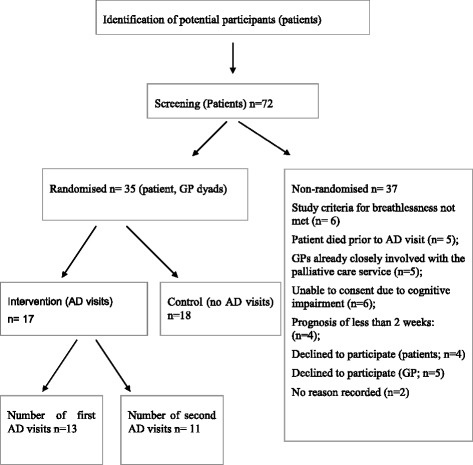
Study flow chart

In total, 72 patients were screened. A total of 35 patient/GP dyads were randomised to the study, 17 to AD visits. The remaining patient/GP dyads (37) were excluded because: patients died prior to an AD visit occurring (5); the GPs were already closely involved with the palliative care service (5); patients were deemed by the research nurse not to be able to consent due to cognitive impairment (6); and patients had a poor life-expectancy (prognosis of less than 2 weeks) (4); Six patients did not meet the study criteria for breathlessness. Three patients declined to participate in the study stating “feeling too unwell” and one patient declined stating “too burdensome” as the reason. Five GPs declined the offer to participate (stating time constraints as the primary reason); No reason was recorded for two GP/patient dyads [Fig. 1].

#### Acceptability

Most GPs randomised to the AD intervention agreed to participate in the AD visits. All GPs but one in the intervention group who completed the questionnaire (n =10), reported that they found the visits useful for their practice [Table 1].

**Table 1:** Qualitative feedback from general practitioners participating in academic detailing provided by a consultant physician on the assessment and treatment of chronic refractory breathlessness

GP (AD group) comments
I am very keen for my patients to be involved in trials as they learn more about their condition, get the benefit of latest ideas, and as a result I learn from this.
Quick, informative. I was unaware of the benefit of morphine for cancer related breathlessness
It was probably excellent for some practices
Well-done
Very good to have one to one discussion. I already had book but didn’t know about web resources
Limited usefulness
Informative, practical and helpful
Reinforcing the need to exclude reversible causes. Tricks on commencing morphine for dyspnoea in patients already on opioids.
Reinforcing current knowledge
Clarified use of narcotics; refined use of nebulised medications

The majority of GPs in the control group who completed the questionnaire (n = 15) requested a copy of the supporting written materials.

#### Feasibility

As noted, five patients died before an AD visit could occur. Most visits to GPs’ offices could be timetabled reasonably easily although difficulty doing this was the major reason for visits not occurring. Thirteen first visits and 11 s visits occurred.

#### Academic detailing visits

In the intervention group, GPs found AD visits helpful. All but one GP in the intervention group reported that the AD materials covered the clinical area of breathlessness and were useful to their practice. All but two GPs in the intervention group reported an increase in their knowledge and confidence in the management of breathlessness. The AD session resulted in 58 % of GPs in the intervention group reporting a change in their approach to the management of breathlessness.

Conversely, 81 % of the usual care group reported low confidence in the management and knowledge of breathlessness before the detailing material was provided to them at the end of the study.

#### Time invested

The duration of visits ranged between 15 and 45 min. Adding travel time to this brought the total time per visit to between 60 and 90 min. Administrative time in organising each visit required approximately 30 min. Total direct costs in today’s terms that include general practitioner, consultant physician and administrative time brought each visit to between AU$290 and AU$435.

## Discussion

This randomised, pilot study provided data to assess the feasibility, acceptability and changes in knowledge and behaviours in response to AD on the diagnosis and management of refractory breathlessness relating to specific patients delivered by a local respiratory physician to GPs. Given the burden of refractory breathlessness in the community, these findings suggest that a fully powered study is justified given the rapidly evolving evidence base in safely treating refractory breathlessness at the end of life.

Patients with multi-morbidities and considerable symptom burden are the largest users of health services [24] and require careful clinical management [25]. Despite this, clinical guidelines most often provide guidance related to single aetiology and deal poorly with multi-morbidity, therefore not reflecting the complexity of patients GPs see in their daily practice.

This is the only study we can identify that uses a consultant physician to detail general practitioners. A recent study adopted AD to improve end-of-life care in the ICU setting [26]. However, AD was only one of five components of the interventions that included: education of clinicians about palliative care using a variety of approaches and training of champions. In the ICU study, the intervention had no measurable effect on ratings of the quality of dying or family satisfaction. However, it is not clear how the academic detailing was conducted [26].

This pilot study, by contrast, utilised well-established AD principles. Results illustrate the potential of targeted AD by a specialist consultant to GPs, enhancing their knowledge of diagnosis of reversible causes and management and confidence in dealing with symptomatic breathlessness for specific patients. Establishing the feasibility and acceptability of AD by a local specialist to GPs in the care of people with breathlessness represents an important step in advancing AD as a strategy for improving the safety and quality of clinical management for this population.

The value GPs placed on AD in this study may be associated with having a detailer with extensive credible clinical experience and expertise as both a palliative and respiratory physician who was able to integrate the specific key messages with a patient specific discussion, and who was known and trusted by the local GP community. As health care systems in resource rich countries face the prospect of the ageing population and caring for people with multiple morbidities and their associated symptoms, the resources of specialist services will come under increasing resource pressure. Building the capacity of the existing workforce to adopt new evidence as it emerges using innovative and cost effective strategies will be critical. Palliative care skills are often best acquired when facilitated in an experiential format [27]. Academic detailing by a local specialist may provide one strategy to facilitate practice change where the behavioural goal relates to increasing diagnostic skills and confidence to support best practice palliative clinical management.

### Limitations

The main limitation of the study was the ultimate change for patients was not measured, but this will be a specific outcome measure in the fully powered study. While GPs rated the visits from the specialist positively, it is unknown if these visits translated into behaviour change and improved clinical outcomes. This will be a crucial outcome in a subsequent adequately powered study. AD is associated with significant upfront costs but is a very effective intervention, limited most frequently by the ability to timetable visits.

## Conclusions

Overall the delivery of academic detailing by a specialist was valued by GPs in terms of acceptability and feasibility. AD may have an important role in the translation of evidence-to-practice in the diagnosis and clinical management of refractory breathlessness and other symptoms at the end of life. Further research is required to determine if academic detailing translates into improved patient outcomes in the palliative care setting in managing refractory breathlessness. This pilot supports a fully powered study.
